# Polycystic ovary syndrome in Salvador, Brazil: a prevalence study in primary healthcare

**DOI:** 10.1186/1477-7827-10-96

**Published:** 2012-11-22

**Authors:** Ligia Gabrielli, Estela ML Aquino

**Affiliations:** 1 Centro de Diabetes e Endocrinologia da Bahia, Av. ACM, s/n, Iguatemi, 40275-350, Salvador, Brazil; 2 Instituto de Saúde Coletiva, Universidade Federal da Bahia, Rua Basílio da Gama, s/n, Campus Universitário do Canela, 40110-040, Salvador, BA, Brazil; 3 MUSA – Programa de Estudos de Gênero e Saúde, Instituto de Saúde Coletiva, Rua Basílio da Gama, s/n, Campus Universitário do Canela, 40110-040, Salvador, BA, Brazil

**Keywords:** Cross-sectional studies, Hyperandrogenism, Polycystic ovary syndrome, Reproductive medicine, Women’s health

## Abstract

**Background:**

Polycystic ovary syndrome (PCOS) is a common condition in women of reproductive age. It is characterized by hyperandrogenism, oligomenorrhea/amenorrhea and polycystic ovaries. It is associated with obesity, diabetes, dyslipidemia and cardiovascular disease. No studies have been conducted on the prevalence of PCOS in Brazilian or South American women. Few studies using the Rotterdam criteria have been published. The objective of the present study was to calculate the prevalence of PCOS at primary healthcare level in Salvador, Brazil based on these criteria.

**Methods:**

This was a cross-sectional, two-phase study conducted in a probability sample of women of 18–45 years of age screened for cervical cancer in the primary healthcare network of the city of Salvador, Brazil. In the first phase, interviews were conducted, weight, height, waist circumference, blood pressure and random blood sugar levels were measured, and the presence of acne and hirsutism was investigated. Women with at least one diagnostic criterion were referred for the second phase, which consisted of specialist consultation, pelvic ultrasonography and hormone measurements for differential diagnosis and/or investigation of a second criterion.

**Results:**

Of the 859 women interviewed, 88.5% were black and 58.7% had 11 years of schooling or less. A diagnosis of PCOS was excluded in 84.4%, undetermined in 7.1% and confirmed in 8.5% (95%CI: 6.80–10.56). There were no statistically significant differences between these three groups with respect to weight, body mass index, waist circumference, blood sugar levels or arterial blood pressure. Women with PCOS were younger (p = 0.00), taller (p = 0.04), had fewer children (p = 0.00), were better educated (p = 0.01), and had higher total testosterone levels (p = 0.01) and a higher LH/FSH ratio (p = 0.01).

**Conclusion:**

According to the Rotterdam criteria, the prevalence of PCOS in women seeking primary healthcare in Salvador, Brazil, was 8.5%.

## Background

Polycystic ovary syndrome (PCOS) is characterized by anovulation, hyperandrogenic symptoms and infertility [1-3]. PCOS is a hyperinsulinemic syndrome strongly associated with obesity and type 2 diabetes mellitus and has also been associated with high blood pressure, dyslipidemia and cardiovascular disease [2]. It is the most common endocrinopathy in women of reproductive age and is identifiable in early adolescence [4-6].

PCOS must be diagnosed as early as possible in order to prevent and treat associated conditions and ultimately reduce cardiovascular morbidity and mortality [7,8]. Nevertheless, the disorder is commonly underdiagnosed, making it impossible to promote preventive measures such as dietary control, physical activity and, whenever required, pharmacological treatment for insulin resistance and associated diseases [9,10]. The heterogeneity in the clinical signs of PCOS and the lack of uniformity in its defining symptoms hamper diagnosis. In recent years, specialists have attempted to reach a consensus regarding the definition of diagnostic criteria. In 1990, the National Institutes of Health (NIH) established that a diagnosis of PCOS required the simultaneous presence of two clinical criteria: oligoovulation or anovulation and clinical and/or biochemical hyperandrogenism, after all other possible etiologies had been excluded. In 2003, the Rotterdam Consensus (RC), sponsored by the European Society for Human Reproduction and Embryology and the American Society for Reproductive Medicine, stipulated that at least two of the following three criteria should be present: oligoovulation or anovulation, clinical and/or biochemical hyperandrogenism and ultrasonographically detected polycystic ovaries, after the exclusion of all other possible etiologies [11]. The criterion concerning ovarian morphology was added to the initial diagnostic criteria; however, it is absent in 5–20% of cases [10,12]. The inclusion of phenotypes unrelated to hyperinsulinism remains under debate. Some authors argue that PCOS is a primarily hyperandrogenic condition; therefore, phenotypes that do not include this feature should not be accepted as such [13]. The presence of some of the relevant clinical aspects or all of them appears to be related to a greater or lesser degree to insulin resistance and to the metabolic syndrome [13,14], which may determine prognosis.

Until a few years ago, the prevalence studies conducted on PCOS were restricted to the United States and some European countries, with reported rates that ranged from 4.6 to 8.0% [6,14-18]. This variation may be explained by differences in methodology and in the age-group and ethnicity of the sample populations [19].

Investigators from developing countries with diverse ethnic populations were encouraged to conduct their own evaluations. In July 2008, two studies on the prevalence of PCOS were published simultaneously: a Sri Lankan population-based survey that used the RC criteria to evaluate 2,915 women and reported a prevalence of 6.3% [10], and a Chinese study that evaluated 915 women attending a healthcare unit and reported a prevalence of 2.2% using the NIH criteria [12]. In a Mexican study published in 2010, a prevalence of PCOS of 6.0% was reported in a voluntary population of 150 women in Mexico City [20]. This finding differs from an earlier estimate of 12.8% in Mexican-American women [21]. A recent publication showed an estimated prevalence of 14.6% in Iranian women evaluated in accordance with the Rotterdam criteria [22].

The objective of the present study was to estimate the prevalence of PCOS in Salvador, Brazil. It was expected that the ensuing results would enable clinical protocols to be created to detect the disorder at an early stage at primary healthcare level.

## Methods

### Design, study population and sample

This cross-sectional study was carried out in the city of Salvador, Brazil with women of 18–45 years of age attending primary healthcare units for cervical cancer screening in all the sanitary districts of the city. This population was selected because screening is aimed at “healthy” women, neither selecting nor excluding any woman in the age-group of interest. Stratified sampling [23] was adopted according to the sanitary district, making a total of 12 strata, with one randomly selected unit in each district. The work shifts during which the subjects would be approached were also chosen at random by elaborating a schedule of preestablished dates on which to conduct the study at each center.

The calculated sample size (n = 850) was distributed proportionally in accordance with the total number of consultations conducted previously in each district. The STATA software program, version 9.0 (Stata Corp., College Station, Texas, USA) was used to calculate sample size and randomize sample units.

The age-group was selected to include the reproductive years. The lower limit was established at 18 years, since this is the age of legal majority, thereby making it simpler to obtain participants’ informed consent. The upper limit was set at 45 years of age despite the frequent occurrence of menopause-related menstrual disorders in women over 40 years of age that could be confused with PCOS symptoms. Nevertheless, FSH levels were measured in all women with menstrual dysfunction to identify any menopausal women. All participants with FSH levels > 25 mIU/ml were excluded from the study, as were those taking any exogenous sex steroid hormones as contraception or hormone therapy.

#### Phase 1: Screening at primary healthcare level

This project was previously approved by the relevant municipal health authorities and by the internal review board of the Bahia State Referral Center for Diabetes and Endocrinology (CEDEBA) (SISNEP # 156299). Written informed consent was obtained from all the participants of this study.

Pregnant or nursing women and those with cognitive and/or physical limitations that prevented them from answering the questionnaire were excluded from the study.

The questionnaire was pretested at a gynecological endocrinology outpatient clinic (CEDEBA) and a pilot study was conducted with users of a primary healthcare unit that did not participate in the study.

A structured questionnaire was applied face-to-face to obtain information on the identification of the patients as well as their sociodemographic data, information on their pubertal, menstrual, sexual and reproductive history, relevant medical history, prior diagnosis of PCOS, any related treatment and the presence of any hyperandrogenic symptoms. Immediately after this interview, an independent examination was made to verify the presence of hirsutism, significant acne or alopecia. Hirsutism was rated according to the modified Ferriman-Gallwey (FG) scoring system. To standardize findings all participants were evaluated by the same investigator.

To screen for PCOS in the primary healthcare units, women with a history of amenorrhea/oligomenorrhea (defined as an intermenstrual interval ≥35 days) or abnormal uterine bleeding (AUB) associated with anovulation (defined as genital bleeding of 10 days or more at irregular intervals of less than 25 days and no other possible cause, as confirmed at the consultation in phase 2 of the study) were identified from their answers to the questionnaire. Women were considered hirsute when they scored ≥ 6 points on the FG scale [24,25]. Clinical hyperandrogenism was also identified from the presence of moderate to severe acne scoring 3 or 4 in the Investigator’s Global Assessment (IGA) and defined by the presence of frequent noninflammatory comedones and a varying number of papulopustular or nodular lesions with inflammatory characteristics [26].

Weight, height, arterial blood pressure, waist circumference and random blood sugar levels were measured in all participants at the primary healthcare units using standardized methodology. Quality control was carried out by systematically retesting around 10% of participants.

#### Phase 2: Specialist consultation at secondary healthcare level

Women with one or more clinical criteria or a prior diagnosis of PCOS, even in the absence of any signs or symptoms, were referred for the second phase of the study to be carried out at the gynecological endocrinology outpatient department, where one or more specialist consultations were provided. Fasting blood samples were taken from all the participants to measure FSH, LH, TSH, PRL and total testosterone (T) levels using standardized chemiluminescent immunometric assays with the Immulite 1000 immunoassay system (Diagnostic Products Corporation, Los Angeles, USA) and 17α-hydroxyprogesterone by radioimmunoassay (Bio Source Europe S.A., Nivelles, Belgium). Total T levels were considered high when they were above the 95^th^ percentile of the women evaluated and classified as normal at the end of the investigation. For budgetary reasons, ovarian morphology was evaluated by ultrasonography only in women who were found to have at least one criterion in the first phase of the study and who went on to participate in the second phase, and for the sole purpose of seeking a second diagnostic criterion or excluding other causes of AUB. The same specialist performed ultrasonography in all cases. Ovaries were defined as polycystic when ≥12 follicles measuring 2–9 mm in diameter and/or follicles with a volume >10 cm^3^ were found (12). An Aloka Pro-Sound ultrasound scanner, model SSD-5500 PHD (Aloka Corporation Ltd., Tokyo, Japan) was used for the study.

A diagnosis of PCOS was given to all the women who fulfilled the diagnostic criteria defined in the RC, i.e. when two of the three clinical criteria were present (oligomenorrhea/amenorrhea, clinical or biochemical hyperandrogenism and ultrasonographically detected polycystic ovaries) and after all other possible etiologies such as hyperprolactinemia, thyroid dysfunction, nonclassic adrenal hyperplasia (NCAH) and Cushing’s syndrome had been excluded [11].

#### Statistical analysis

The prevalence of PCOS in the study population was calculated with a 95% confidence interval (95%CI) according to the method recommended by Agresti and Coull [27]. In the bivariate analysis, the different sociodemographic, clinical and biochemical characteristics of the women with PCOS were compared with those of the women in whom PCOS was undetermined and those without PCOS. To verify the null hypothesis of equality between the groups of women with and without PCOS, Student’s *t*-test was used to compare means, the Mann–Whitney test for medians and Pearson’s chi-square test for proportions. Gradients were evaluated using Cuzick’s test for trend [28]. Statistical analysis was performed using the Stata software program, version 9.0 (Statcorp, College Station, USA).

## Results

Of all the eligible women screened for the study, 859 were interviewed, yielding a response rate of 96.1%. The mean age of the subjects was 30.7 ± 7.2 years, 88.5% were black (according to the ethnic classifications of the Brazilian Institute of Geography and Statistics) and 62.8% were in a stable marital relationship. The proportion of women in paid employment at the time of the interview was 54.5% (n = 468). Of these, 75.2% (n = 352) earned one minimum wage or less and 95.3% (n = 446) earned a maximum of two minimum wages.

**Figure 1:**
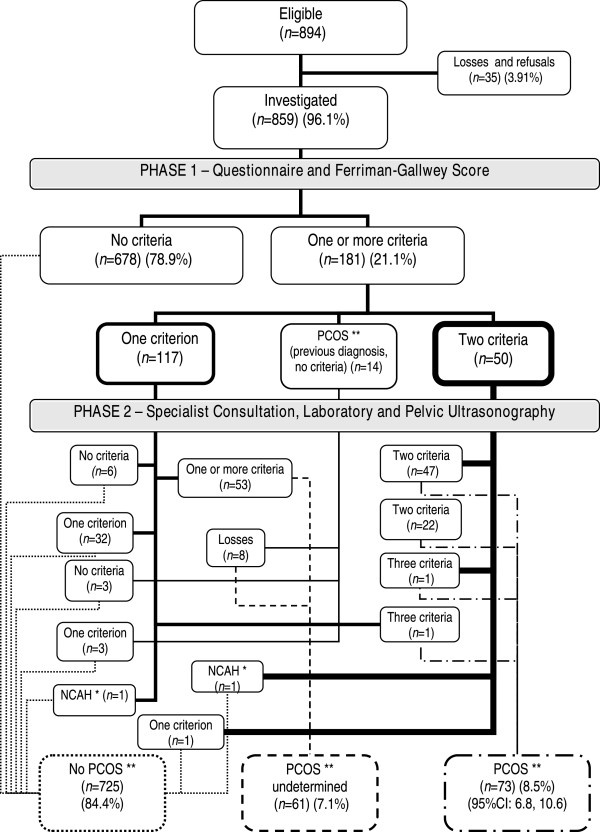
**Flowchart of the study population.** Flowchart of the study population from the moment of screening in the primary healthcare units to further diagnostic assessment at the specialist consultation and referral for supplementary tests, concluding with the final classification. Study on the prevalence of polycystic ovary syndrome at primary healthcare level in Salvador, Brazil, 2008 (n = 859). * Non-classic adrenal hyperplasia. ** Polycystic ovary syndrome.

Of all women in the study, 181 (21.1%) were classified as having one (13.6%) or two (5.8%) diagnostic criteria or, alternately, having previously been diagnosed with PCOS despite having no clinical criteria whatsoever (1.6%). These women were then referred to the second phase of the trial (Figure 1) and 107 (59.1%) attended the specialist consultation. Of these, 91.6% underwent hormone evaluation and 57.9% were referred for ultrasonography, with a compliance rate of 90.3%. Of those who failed to attend the specialist visit, 24.7% already had two criteria, thus constituting a diagnosis of PCOS.

A total of 73 women fulfilled the prerequisites for a diagnosis of PCOS according to the RC criteria, resulting in an estimated prevalence of 8.5% (95%CI: 6.80 - 10.56). When the NIH diagnostic criteria were applied, 69 cases of PCOS were found, with a prevalence of 8.03% (95%CI: 6.39 - 10.05). Of the 859 women participating in the study, 84.4% were considered not to have PCOS. In those women with only one criterion who failed to attend the specialist consultation in the second phase of the study or who attended the consultation but failed to complete the evaluation, PCOS was considered undetermined (7.1%) (Figure 1).

When comparing measurements between the three groups of women in whom PCOS was confirmed, undetermined or absent, gradients were found between the groups with respect to all the measurements that attained statistical significance, indicating that there were probably some cases of PCOS in the undetermined group that remained undetected. In the extreme possibility that all the women classified as undetermined actually had PCOS, the maximum prevalence would be 15.6% (95%CI: 13.32 - 18.18).

**Figure 2:**
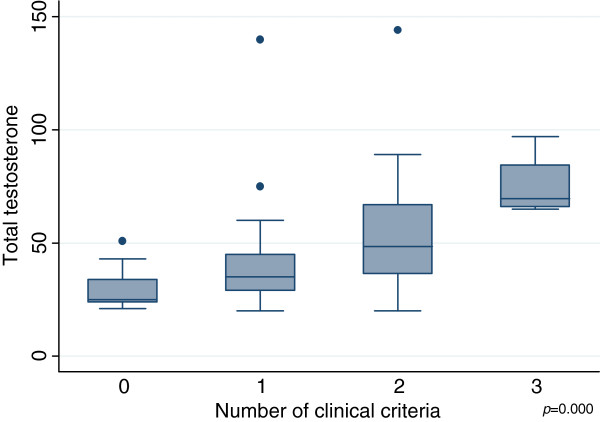
Testosterone levels according to the number of clinical criteria.

**Figure 3:**
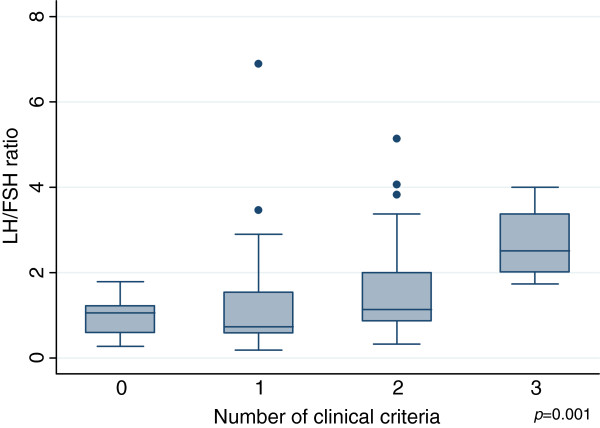
LH/FSH ratio according to the number of clinical criteria.

No statistically significant differences were found between the groups with regard to weight, body mass index, waist circumference, random blood sugar levels or minimum, maximum or mean arterial blood pressure (Table 1). Women with PCOS were younger (p = 0.00), taller (p = 0.04), had fewer children (p = 0.00) and more years of schooling (p = 0.01). Following adjustment for age, the differences in all of these variables remained statistically significant except for height (p = 0.09) (Table 2). With regard to the number of years of schooling, when this variable was categorized into four education levels, there was a clear trend towards a better education level in the group of women diagnosed as having PCOS (p = 0.00) (Table 3). Total testosterone levels were higher in this group (p = 0.01), with a gradient that increased according to the number of criteria present (p = 0.00) (Figure 2). The LH/FSH ratio was also higher in women with PCOS (p = 0.01) and the gradient was similar to that of testosterone with respect to the number of criteria present (p = 0.00) (Figure 3).

**Table 1:** Sociodemographic, anthropometric, clinical and biochemical characteristics of women participating in a study on the prevalence of PCOS, Salvador, Brazil, 2008 (n = 859)

***Variables***	**PCOS Confirmed**	**PCOS Undetermined**	**Non-PCOS**	**Statistical Test**^**b**^
	**(*n *=73)**	^**a**^**(*n *=61)**	**(*n *=725)**	
	Mean (SD) ^c^	Mean (SD)	Mean (SD)	Student’s *t*-test
Age	28.4 (6.5)	29.3 (7.3)	31.0 (7.3)	0.00
Number of children	0.9 (1.1)	1.2 (1.4)	1.5 (1.3)	0.00
Years of schooling	9.7 (2.7)	8.2 (3.0)	8.6 (2.9)	0.00
Height (meters)	1.61 (0.07)	1.60 (0.05)	1.59 (0.06)	0.04
	Median (IQR) ^d^	Median (IQR)	Median (IQR)	Mann–Whitney test
*Measurements*				
Weight (kg)	60.9 (37.7 – 84.1)	60.8 (44.3 – 77.3)	61.1 (45.1 – 77.1)	0.50
Body mass index (kg/m^2^)	24.2 (17.7 – 30.7)	23.7 (18.1 – 29.3)	24.1 (18.1 – 30.1)	0.92
Waist circumference (cm)	74 (56 – 92)	74 (56 – 92)	75 (60 – 90)	0.60
Random blood sugar ^e^	92 (71 – 114)	93 (71 – 115)	92 (72 – 112)	0.67
Systolic blood pressure (mmHg)	119 (99 – 139)	118 (96 – 140)	122 (102 – 142)	0.31
Diastolic blood pressure (mmHg)	74 (60 – 88)	74 (59 – 89)	74 (59.5 – 89.5)	0.92
Mean arterial pressure (mmHg)	97.5 (81 – 114)	96.5 (79 – 114)	98.5 (81 – 116)	0.48
Total testosterone ^f^	52 (20 – 82)	NA ^g^	35 (16 – 54)	0.00
LH/FSH ratio ^h^	1.31 (−0.15 – 2.77)	1.04 (−3.16 – 5.24)	0.82 (−0.1 – 1.76)	0.01
	(%)	(%)	(%)	*χ*^2^
*PCOS Diagnostic Criteria*				
Oligomenorrhea ^i^	98.6	34.4	2.5	0.00
Hirsutism ^j^	79.5	47.5	3.1	0.00
Total testosterone ≥ 95^th^ percentile ^k^	44.0	25.0	14.3	0.00
Polycystic Ovaries ^l^	15.1 ^m^	NA ^n^	—	0.01
*Sociodemographic Characteristics*				
Race/skin color/ethnic group				
Black	90.8	91.2	88.1	0.65
Education (years of schooling)				0.01
Did not complete primary school (<8)	16.4	39.3	29.4	
Completed primary school (= 8)	21.9	18.0	28.9	
Completed secondary school (= 11)	53.4	41.0	39.6	
University (>11)	8.22	1.64	2.1	
Marital status				
Stable relationship	65.8	55.7	63.0	0.45
Employment status				
In paid employment	43.8	55.7	55.4	0.16
*Associated Clinical Conditions*				
Blood pressure ≥ 140/90 mm Hg	17.8	18.6	19.1	0.96
Obesity (BMI ^o^ ≥ 30 kg/m^2^)	13.7	18.6	12.3	0.37
Random blood sugar ≥ 100 mg/dl	32.9	39.3	33.4	0.66

^a^ Polycystic Ovary Syndrome according to the criteria defined in the Rotterdam Consensus. ^b^ Statistical tests comparing “confirmed PCOS” with “non-PCOS”. ^c^ Standard deviation. ^d^ Interquartile range. ^e^ Random blood sugar in mg/dl. ^f^ Total serum testosterone (ng/dl). ^g^ Not available (undetermined). ^h^ LH and FSH in mIU/ml. ^i^ Menstrual cycles ≥ 35 days or anovulatory dysfunctional uterine bleeding. ^j^ Ferriman-Gallwey (FG) score ≥ 6. ^k^ Total serum testosterone ≥ the 95th percentile of women with no diagnostic criteria (58 ng/dl). ^l^ By pelvic ultrasonography according to the imaging criteria defined in the Rotterdam Consensus, 2003. ^m^ Performed only to search for a second criterion. ^n^ Not available. ^o^ Body mass index.

**Table 2:** **Characteristics adjusted for age in women participating in a study on the prevalence of PCOS**^**a**^**, Salvador, Brazil, 2008 (n = 859)**

**Variables**	**PCOS (*n *=73)**	**Non-PCOS (*n *=725)**	**Student’s *t*-test**
	**Mean (SD)**^**b**^	**Mean (SD)**	
Number of children	0.49 (0.14)	0.93 (0.64)	0.00
Years of schooling	10.10 (0.34)	9.20 (0.15)	0.01
Height (meters)	1.62 (0.01)	1.60 (0.00)	0.09

^a^ Polycystic Ovary Syndrome.

^b^ Standard deviation.

**Table 3:** **Education level adjusted for age in women with PCOS**^**a **^**participating in a study on the prevalence of PCOS, Salvador, Brazil, 2008 (n = 859)**

	**PR**^**b**^**crude (95%CI)**	**PR adjusted (95%CI)**^**c**^
Education (years of schooling)		
Did not complete primary school (<8)	0.56 (0.31 - 1.00)	0.61 (0.34 - 1.09)
Completed primary school (=8)	0.76 (0.46 - 1.26)	0.73 (0.44 - 1.22)
Completed secondary school (=11)	1.35 (0.96 - 1.86)	1.30 (0.93 - 1.82)
University (>11)	3.97 (1.54 - 10.22)	4.11 (1.58 - 10.67)

^a^ Polycystic Ovary Syndrome.

^b^ Prevalence ratio.

^c^ Cuzick’s trend test p = 0.00.

The main characteristics of the women screened in the first phase of the study were oligomenorrhea/amenorrhea (54.7%) and hirsutism (59.7%) (Table 4). Likewise, at the end of the study, 78.1% of the 73 participants who had been classified as having PCOS were found to have a combination of these two clinical criteria. Of these, 96.3% had oligomenorrhea and 79.5% had hirsutism. Serum testosterone measurements added another eight cases (11%). Taking acne into consideration for the diagnosis of PCOS and performing ultrasonography in women with only one criterion identified another four cases each (5.5%).

**Table 4:** Characteristics of women in the different phases of the study on the prevalence of PCOS, Salvador, Brazil, 2008 (n = 859)

**Phase 1 – Characteristics (*n***_**1**_**=181)**	***n***	**%**	**Phase 2 - Phenotypes (*n***_**2**_**=73)**	***n***	**%**
Oligomenorrhea/amenorrhea	99	54.7	Oligo/amenorrhea and hirsutism ^b^	57	78.1
Hirsutism ^b^	108	59.7	Oligo/amenorrhea and high testosterone levels ^d^	8	10.9
Moderate to severe acne ^c^	21	11.6	Oligo/amenorrhea and acne ^c^	4	5.5
Abnormal uterine bleeding	5	2.8	Oligo/amenorrhea and polycystic ovaries ^a^	3	4.1
Androgenic alopecia	3	1.7	Hirsutism and polycystic ovaries	1	1.4

^a^Rotterdam Consensus Criteria ^b^Ferriman-Gallwey score ≥ 6 ^c^Moderate to severe forms (Investigator’s Static Global Assessment) ^d^ ≥ 95 ^th^ percentile of women with no clinical criteria *n*_1_ = number of women with at least one criterion *n*_2_ = number of women with at least two criteria.

Prevalence rates of 12.6% and 12.9% were found for hirsutism (FG ≥ 6) and for oligomenorrhea/amenorrhea/AUB, respectively. Overall, 18.5% of the women in the study had acne, which was classified as moderate to severe in 2.5% of these cases. In addition, 0.4% of women had androgenic alopecia. PCOS was confirmed in one-third of the women with more severe acne and in two-thirds of those with alopecia.

Of the 126 women who declared in the questionnaire that they had hirsutism and the 196 who stated that they had acne, 108 and 157 women, respectively, had these diagnoses confirmed by the FG score or by dermatological examination (sensitivity of 55.6% and specificity of 91.1% for hirsutism and sensitivity of 51.6% and specificity of 83.4% for acne).

## Discussion

The present results showed a prevalence of PCOS of 8.5% (95%CI: 6.80 - 10.56) in accordance with the Rotterdam criteria in users of the primary public healthcare service in the city of Salvador. When this finding was compared with the prevalence obtained using the NIH criteria (8.03%; 95%CI: 6.39 - 10.05), no statistically significant difference was found, as shown by the overlapping confidence intervals.

Studies conducted in the southern USA [6,15], Greece [17] and Spain [18] reported prevalence rates of 4.6 - 6.8% using the NIH criteria. The measurements of occurrence were expected to increase between 39% and 50% with the use of the RC, since these criteria may increase the likelihood of diagnosis [29,30]. The South Asian study conducted in Sri Lanka and the Mexican study reported unexpectedly low prevalence rates of 6.3% and 6.6% (95%CI: 2.3 - 10.9) despite using the RC criteria [10,20]. In agreement with the Iranian study, the present study showed a higher prevalence, as expected for the criteria that were used [21]. It is important to emphasize that the results found in the various studies would be different if other cut-off points on the FG scale had been used and in this respect the data are not comparable. In the present study, a more sensitive cut-off point (FG ≥ 6) was selected, which led to the identification of a greater number of cases. This was also the choice of most of the nine principal studies on the prevalence of PCOS published to date, in which four used a cut-off point of FG ≥ 6 [6,12,15,17], three used FG ≥ 8 [18,20,22] and one used FG ≥ 7 [16]. In the remaining study, self-reported hirsutism was the criterion used [14].

This study included almost all diagnostic possibilities with the exception of the phenotype represented by polycystic ovaries and hyperandrogenemia for which the methodology used for screening was not applicable. To optimize available resources, testosterone measurements and ultrasound scans were not performed in all the women included in the study. It was decided to ignore these possible cases since they are less severe [31] and less common. Their occurrence has been estimated at 0.6% in population-based samples [10] and 5.8% in selected samples [32]. In the present study, the free androgen index was not calculated and for operational reasons progesterone was not measured to confirm anovulation. It was decided that all women reporting oligomenorrhea or amenorrhea would be considered anovulatory [3].

### Methodological challenges in achieving comparable data

This study highlights the difficulties involved in obtaining information on hirsutism and acne through the use of a questionnaire, revealing the poor sensitivity of this method compared to medical consultation or examination. According to the answers given in the questionnaire, 44% fewer cases of hirsutism and 48% fewer cases of acne were identified compared to those found when the FG score or skin examination were used, both of which were performed in the entire study population. Since body image is constructed as a function of social concepts, what is considered to constitute “excess” body hair is profoundly influenced by cultural elements in a given population. Therefore, response to a questionnaire on these aspects is obligatorily loaded with individualized symbolic meanings that are difficult to standardize. Ethnic diversity in itself brings cultural diversity that is often reflected in the way one sees one’s own body, including skin characteristics and hair distribution. In the case of hirsutism, it would therefore be recommendable to carry out further analysis using an anthropological approach on the series of symbolic meanings associated with the answers to the study questionnaires in order to attempt to incorporate cultural aspects when formulating questions.

Ethnic differences must be taken into consideration when analyzing studies on PCOS. In a Chinese article, no cases of hirsutism were found among 915 women [12]. Other investigators have already reported a low prevalence of hirsutism in Asian women [33,34]. On the other hand, a high proportion of hirsutism has been found in Greek women [17]. Ethnic variations in the occurrence of hirsutism reflect one of the difficulties in standardizing diagnostic criteria for PCOS and the consequent need for local studies.

The rates of oligomenorrhea/amenorrhea reported in previous studies on PCOS vary greatly, ranging from 11.9% to 46.5%. The figure found in this study is among the lowest found (12.9%).

Mean total testosterone levels and the mean LH/FSH ratio increased in a progressive gradient as a function of the number of clinical criteria presented by the participants. These data are consistent with the findings of Kumarapeli et al., who reported the lowest testosterone levels in controls followed by probable cases (one criterion) and the highest levels in confirmed PCOS cases (at least two criteria) [11]. Chen et al. evaluated the clinical aspects of PCOS and reported higher LH/FSH ratios in women with two criteria compared to those with just one [12].

In the present study, a positive correlation was found between PCOS and height; however, statistical significance was lost following adjustment for age (Table 2), since the participants with PCOS were younger and it has already been shown that there is a tendency towards increased stature in younger generations [35]. Nevertheless, in an earlier publication, Dessel et al. reported higher serum levels of the free fraction of insulin growth factor (IGF-1) in women with PCOS compared to a control group and lower levels of IGF-1 binding protein (IGFBP-1) [36]. Garcia-Rudaz et al. reported regular pulsatility patterns and higher levels of growth hormone secretion in non-obese women with PCOS, similar to those found in males [37]. These findings permit speculation that these biochemical modifications may increase the stature of women with PCOS. Future studies should be designed for the specific purpose of testing this hypothesis.

### PCOS in Brazil and its sociocultural aspects

This is the first study on the prevalence of PCOS to be conducted either in Brazil or South America. Using the criteria defined in the RC, the methodology applied was shown to be appropriate for the screening and diagnosis of this syndrome in primary healthcare services, opening a gateway to the possibility of implementing clinical protocols to identify cases of PCOS at primary healthcare level.

The objective of this study was not to examine the potential effects of the vast socioeconomic differences that exist in Salvador on the occurrence of PCOS, since the study was conducted exclusively among adult female users of the public healthcare network. Women who do not use the healthcare services, those attending outsourced services of the National Health System and women who use supplementary or private healthcare services were not included in this sample. The latter group represents the most economically affluent sector of the population and is a minority. Although this sample is limited, it is fairly representative of the population of Salvador with regard to ethnic composition, education level and income [38,39].

Nevertheless, this apparent homogeneity harbors a surprising finding: the higher education level of the women with PCOS compared to controls, even when adjusted for age (Table 2). There was also a positive trend for better education levels within the PCOS group (p = 0.00) (Table 3). A possible explanation for this association may lie in the fact that women with PCOS have fewer children, which may have given them a better opportunity to advance their education. This would confirm reports of an inverse relationship between schooling and parity that has been widely reported in the literature. Furthermore, a study carried out in Brazil [40] confirms that schooling is associated with greater care in reproductive life, later sexual debut and fewer children, possibly explaining part of the effect found in the present study. Exploring socioeconomic status in women with PCOS, Merkin et al. recently found that the risk of the syndrome and its components is higher in the presence of a combined effect of poor parental education/high personal education [41].

## Conclusions

This was the first study conducted in Brazil or South America to estimate the prevalence of PCOS in a random population. The prevalence rate found was 8.5% (95%CI: 6.80 - 10.56). This paper also describes some interesting and unusual findings such as higher stature and a higher education level in these women, which should be addressed in future studies with appropriate designs. The available literature on PCOS emphasizes clinical and physiopathological aspects. Further studies encompassing sociocultural aspects of this syndrome must be carried out, focusing on questions related both to its causality and to its consequences on social trajectories, elements that may enrich the understanding of the phenomenon and its effect on the life of women affected by the syndrome.

## Abbreviations

AUB: Abnormal uterine bleeding; CEDEBA: Bahia State Referral Center for Diabetes and Endocrinology; CI: Confidence interval; FG: Ferriman-Gallwey; FSH: Follicle stimulating hormone; IGA: Investigator’s Global Assessment; IGFBP-1: Insulin growth factor binding protein; IGF-1: Insulin growth factor; LH: Luteinizing hormone; NIH: National Institutes of Health; PCOS: Polycystic ovary syndrome; RC: Rotterdam consensus; TSH: Thyroid-stimulating hormone.

## Competing interests

The authors declare that they have no competing interests.

## Authors’ contributions

LG worked on the conception of the study and on the protocol, coordinated and supervised the fieldwork, carried out the statistical analysis and interpretation of data, and drafted the manuscript. EMLA contributed to the analysis, worked on the discussion of the results, and helped draft the manuscript. Both authors read and approved the final manuscript.

## Authors’ information

Ligia Gabrielli, MD, MPH

Gynecologist, Gynecological Endocrinology Outpatient Clinic, Centro de Diabetes e Endocrinologia da Bahia.

Investigator, Institute of Collective Health, Federal University of Bahia, Brazil.

Estela Maria Leão de Aquino, MD, MPH, DrPH

Professor of Epidemiology; Methods for Health Research; Gender, Sexuality and Reproductive Health at the Institute of Collective Health, Federal University of Bahia, Brazil.

## References

[B1] Legro RS Diagnostic criteria in polycystic ovary syndrome Semin Reprod Med 2003 21 3 267 275 14593549 10.1055/s-2003-43304

[B2] Ehrmann DA Polycystic ovary syndrome N Engl J Med 2005 352 12 1223 1236 10.1056/NEJMra041536 15788499

[B3] Speroff LGR Kase NG Weinberg R Anovulation and the Polycystic Ovary Clinical Gynecologic Endocrinology and Infertility 2005 7 Philadelphia: Lippincott Williams and Wilkins 465 498

[B4] Carmina E Lobo RA Polycystic ovary syndrome (PCOS): arguably the most common endocrinopathy is associated with significant morbidity in women J Clin Endocrinol Metabol 1999 84 6 1897 1899 10.1210/jc.84.6.1897 10372683

[B5] Franks S Polycystic ovary syndrome N Engl J Med 2005 333 13 9 10.1056/NEJM199509283331307 7651477

[B6] Azziz R Woods KS Reyna R Key TJ Knochenhauer ES Yildiz BO The prevalence and features of the polycystic ovary syndrome in an unselected population J Clin Endocrinol Metabol 2004 89 6 2745 2749 10.1210/jc.2003-032046 15181052

[B7] Legro RS Kunselman AR Dodson WC Dunaif A Prevalence and predictors of risk for type 2 diabetes mellitus and impaired glucose tolerance in polycystic ovary syndrome: a prospective, controlled study in 254 affected women J Clin Endocrinol Metabol 1999 84 1 165 169 10.1210/jc.84.1.165 9920077

[B8] Krentz AJ von Muhlen D Barrett-Connor E Searching for polycystic ovary syndrome in postmenopausal women: evidence of a dose-effect association with prevalent cardiovascular disease Menopause 2007 14 2 284 292 10.1097/GME.0b013e31802cc7ab 17245231 PMC2642654

[B9] Sills ES Perloe M Tucker MJ Kaplan CR Genton MG Schattman GL Diagnostic and treatment characteristics of polycystic ovary syndrome: descriptive measurements of patient perception and awareness from 657 confidential self-reports BMC Womens Health 2001 1 1 3 10.1186/1472-6874-1-3 11545683 PMC55341

[B10] Kumarapeli V Seneviratne Rde A Wijeyaratne CN Yapa RM Dodampahala SH A simple screening approach for assessing community prevalence and phenotype of polycystic ovary syndrome in a semi-urban population in Sri Lanka Am J Epidemiol 2008 168 3 321 328 10.1093/aje/kwn137 18550559

[B11] Group PCW Revised 2003 consensus on diagnostic criteria and long-term health risks related to polycystic ovary syndrome Fertil Steril 2004 81 1 19 25 10.1016/j.fertnstert.2003.10.004 14711538

[B12] Chen X Yang D Mo Y Li L Chen Y Huang Y Prevalence of polycystic ovary syndrome in unselected women from southern China Eur J Obstet Gynecol Reprod Biol 2008 139 1 59 64 10.1016/j.ejogrb.2007.12.018 18378061

[B13] Azziz R Carmina E Dewailly D Diamanti-Kandarakis E Escobar-Morreale HF Futterweit W Janssen OE Legro RS Norman RJ Taylor AE Positions statement: criteria for defining polycystic ovary syndrome as a predominantly hyperandrogenic syndrome: an Androgen Excess Society guideline J Clin Endocrinol Metabol 2006 91 11 4237 4245 10.1210/jc.2006-0178 16940456

[B14] Lindholm A Andersson L Eliasson M Bixo M Sundstrom-Poromaa I Prevalence of symptoms associated with polycystic ovary syndrome Int J Gynaecol Obstet Offic Organ Int Fed Gynaecol Obstet 2008 102 1 39 43 10.1016/j.ijgo.2008.01.023 18321516

[B15] Knochenhauer ES Key TJ Kahsar-Miller M Waggoner W Boots LR Azziz R Prevalence of the polycystic ovary syndrome in unselected black and white women of the southeastern United States: a prospective study J Clin Endocrinol Metabol 1998 83 9 3078 3082 10.1210/jc.83.9.3078 9745406

[B16] Michelmore KF Balen AH Dunger DB Vessey MP Polycystic ovaries and associated clinical and biochemical features in young women Clin Endocrinol 1999 51 6 779 786 10.1046/j.1365-2265.1999.00886.x 10619984

[B17] Diamanti-Kandarakis E Kouli CR Bergiele AT Filandra FA Tsianateli TC Spina GG Zapanti ED Bartzis MI A survey of the polycystic ovary syndrome in the Greek island of Lesbos: hormonal and metabolic profile J Clin Endocrinol Metabol 1999 84 11 4006 4011 10.1210/jc.84.11.4006 10566641

[B18] Asuncion M Calvo RM San Millan JL Sancho J Avila S Escobar-Morreale HF A prospective study of the prevalence of the polycystic ovary syndrome in unselected Caucasian women from Spain J Clin Endocrinol Metabol 2000 85 7 2434 2438 10.1210/jc.85.7.2434 10902790

[B19] Li L Yang D Chen X Chen Y Feng S Wang L Clinical and metabolic features of polycystic ovary syndrome Int J Gynecol Obstet 2007 97 2 129 134 10.1016/j.ijgo.2007.01.005 17368460

[B20] Moran C Tena G Moran S Ruiz P Reyna R Duque X Prevalence of polycystic ovary syndrome and related disorders in mexican women Gynecol Obstet Invest 2010 69 4 274 280 10.1159/000277640 20110726

[B21] Goodarzi MO Quinones MJ Azziz R Rotter JI Hsueh WA Yang H Polycystic ovary syndrome in Mexican-Americans: prevalence and association with the severity of insulin resistance Fertil Steril 2005 84 3 766 769 10.1016/j.fertnstert.2005.03.051 16169421

[B22] Tehrani FR Simbar M Tohidi M Hosseinpanah F Azizi F The prevalence of polycystic ovary syndrome in a community sample of Iranian population: Iranian PCOS prevalence study Reprod Biol Endocrinol 2011 9 39 10.1186/1477-7827-9-39 21435276 PMC3070632

[B23] Bolfarine HBW Elementos de Amostragem 2005 1 São Paulo: Edgard Blucher

[B24] Ferriman D Gallwey JD Clinical assessment of body hair growth in women J Clin Endocrinol Metabol 1961 21 1440 1447 10.1210/jcem-21-11-1440 13892577

[B25] Yildiz BO Diagnosis of hyperandrogenism: clinical criteria Best Pract Res Clin Endocrinol Metab 2006 20 2 167 176 10.1016/j.beem.2006.02.004 16772149

[B26] U.S. Department of Health and Human Services, Food and Drug Administration, Center for Drug Evaluation and Research Draft Guidance for Industry: Acne Vulgaris: Developing Drugs for Treatment Clinical/Medical. September 2005. Accessed at http://www.fda.gov/downloads/Drugs/GuidanceComplianceRegulatoryInformation/Guidances/UCM071292.pdf

[B27] Agresti ACB Approximate is better than “Exact” for interval estimation of binomial proportions Am Statist 1998 52 2 8

[B28] Cuzick J A Wilcoxon-type test for trend Stat Med 1985 4 1 87 90 10.1002/sim.4780040112 3992076

[B29] Hsu MI Liou TH Chou SY Chang CY Hsu CS Diagnostic criteria for polycystic ovary syndrome in Taiwanese Chinese women: comparison between Rotterdam 2003 and NIH 1990 Fertil Steril 2007 88 3 727 729 10.1016/j.fertnstert.2006.11.149 17706215

[B30] Broekmans FJ Knauff EA Valkenburg O Laven JS Eijkemans MJ Fauser BC PCOS according to the Rotterdam consensus criteria: Change in prevalence among WHO-II anovulation and association with metabolic factors BJOG 2006 113 10 1210 1217 10.1111/j.1471-0528.2006.01008.x 16972863

[B31] Azziz R Diagnostic criteria for polycystic ovary syndrome: a reappraisal Fertil Steril 2005 83 5 1343 1346 10.1016/j.fertnstert.2005.01.085 15866567

[B32] Diamanti-Kandarakis E Panidis D Unravelling the phenotypic map of polycystic ovary syndrome (PCOS): a prospective study of 634 women with PCOS Clin Endocrinol 2007 67 5 735 742 10.1111/j.1365-2265.2007.02954.x 17760884

[B33] Carmina E Koyama T Chang L Stanczyk FZ Lobo RA Does ethnicity influence the prevalence of adrenal hyperandrogenism and insulin resistance in polycystic ovary syndrome? Am J Obstet Gynecol 1992 167 6 1807 1812 1471702 10.1016/0002-9378(92)91779-a

[B34] Rodin DA Bano G Bland JM Taylor K Nussey SS Polycystic ovaries and associated metabolic abnormalities in Indian subcontinent Asian women Clin Endocrinol 1998 49 1 91 99 10.1046/j.1365-2265.1998.00492.x 9797852

[B35] Kac G Secular height trend: a literature review Cadernos de saude publica/Ministerio da Saude, Fundacao Oswaldo Cruz, Escola Nacional de Saude Publica 1999 15 3 451 461 10.1590/s0102-311x1999000300002 10502141

[B36] Thierry van Dessel HJ Lee PD Faessen G Fauser BC Giudice LC Elevated serum levels of free insulin-like growth factor I in polycystic ovary syndrome J Clin Endocrinol Metabol 1999 84 9 3030 3035 10.1210/jc.84.9.3030 10487660

[B37] Garcia-Rudaz MC Ropelato MG Escobar ME Veldhuis JD Barontini M Amplified and orderly growth hormone secretion characterizes lean adolescents with polycystic ovary syndrome Eur J Endocrinol/Eur Fed Endocr Soc 2002 147 2 207 216 10.1530/eje.0.1470207 12153742

[B38] Prefeitura Municipal de Salvador Secretaria Municipal de Saúde. Diagnóstico de saúde da população negra de Salvador 2006 Bahia: Salvador

[B39] DIEESE. Departamento Intersindical de Estatística e Estudos Socioeconômicos Anuário da Educação Profissional da Bahia: Indicadores – Região Metropolitana de Salvador 2010 São Paulo Accessed at http://dieese.org.br/ped/ssa/pedssa_2010.xml#

[B40] Bozon MHM Heilborn ML AE, Bozon M, Knauth DR Iniciação à sexualidade: modos de socialização, interações de gênero e trajetórias individuais O aprendizado da sexualidade: um estudo sobre reprodução e trajetórias sociais de jovens brasileiros 2006 1 Rio de Janeiro: Fiocruz/Garamond 156 205

[B41] Merkin SS Azziz R Seeman T Calderon-Margalit R Daviglus M Kiefe C Matthews K Sternfeld B Siscovick D Socioeconomic status and polycystic ovary syndrome J Womens Health (Larchmt) 2011 20 3 413 419 10.1089/jwh.2010.2303 21323584 PMC3115419

